# Familial Temperature-Sensitive Auditory Neuropathy: Distinctive Clinical Courses Caused by Variants of the *OTOF* Gene

**DOI:** 10.3389/fcell.2021.732930

**Published:** 2021-10-07

**Authors:** Yi-Ming Zhu, Qi Li, Xue Gao, Yan-Fei Li, You-Li Liu, Pu Dai, Xiang-Ping Li

**Affiliations:** ^1^Department of Otolaryngology-Head and Neck Surgery, Nanfang Hospital, Southern Medical University, Guangzhou, China; ^2^Department of Otolaryngology-Head and Neck Surgery, Lanzhou University Second Hospital, Lanzhou, China; ^3^Department of Otolaryngology, PLA Rocket Force Characteristic Medical Center, Beijing, China; ^4^Department of Otolaryngology-Head and Neck Surgery, Chinese PLA General Hospital, Beijing, China

**Keywords:** temperature sensitive, auditory neuropathy, variant, *OTOF*, otoferlin, genotype phenotype correlation, hearing loss

## Abstract

**Objective:** To investigate the clinical course and genetic etiology of familial temperature-sensitive auditory neuropathy (TSAN), which is a very rare subtype of auditory neuropathy (AN) that involves an elevation of hearing thresholds due to an increase in the core body temperature, and to evaluate the genotype–phenotype correlations in a family with TSAN.

**Methods:** Six members of a non-consanguineous Chinese family, including four siblings complaining of communication difficulties when febrile, were enrolled in this study. The clinical and audiological profiles of the four siblings were fully evaluated during both febrile and afebrile episodes, and the genetic etiology of hearing loss (HL) was explored using next-generation sequencing (NGS) technology. Their parents, who had no complaints of fluctuating HL due to body temperature variation, were enrolled for the genetics portion only.

**Results:** Audiological tests during the patients’ febrile episodes met the classical diagnostic criteria for AN, including mild HL, poor speech discrimination, preserved cochlear microphonics (CMs), and absent auditory brainstem responses (ABRs). Importantly, unlike the pattern observed in previously reported cases of TSAN, the ABRs and electrocochleography (ECochG) signals of our patients improved to normal during afebrile periods. Genetic analysis identified a compound heterozygous variant of the *OTOF* gene (which encodes the otoferlin protein), including one previously reported pathogenic variant, c.5098G > C (p.Glu1700Gln), and one novel variant, c.4882C > A (p.Pro1628Thr). Neither of the identified variants affected the C2 domains related to the main function of otoferlin. Both variants faithfully cosegregated with TSAN within the pedigree, suggesting that *OTOF* is the causative gene of the autosomal recessive trait segregation in this family.

**Conclusion:** The presence of CMs with absent (or markedly abnormal) ABRs is a reliable criterion for diagnosing AN. The severity of the phenotype caused by dysfunctional neurotransmitter release in TSAN may reflect variants that alter the C2 domains of otoferlin. The observations from this study enrich the current understanding of the phenotype and genotype of TSAN and may lay a foundation for further research on its pathogenesis.

## Introduction

Auditory neuropathy (AN), which is a clinical disorder featuring auditory processing dysfunction, is characterized by normal outer hair cell (OHC) function in the cochlea and abnormal neural function at the level of the inner hair cells (IHCs), the cochlear nerve, or their junction. Patients with AN commonly have normal otoacoustic emissions (OAEs) and cochlear microphonics (CMs) but absent or severely abnormal auditory brainstem responses (ABRs). Pure-tone audiometry can vary widely from normal hearing thresholds to profound hearing loss (HL). Acoustic reflexes (ARs) are often absent, and speech recognition is worse than would be predicted from the pure-tone thresholds, particularly in ambient noise (Starr et al., 1996). Although individuals with AN retain OHC function, they usually have impaired neural coding of sound stimuli as a result of lesions involving the auditory nerve itself, the IHCs and/or their synapses or auditory cortex abnormalities (Kaga et al., 1996). Accordingly, the terms “auditory synaptopathy” and “auditory neuropathy” are applied to this disease when it is due to synaptic and neural deficits, respectively (Moser and Starr, 2016).

Among patients with sensorineural HL, AN accounts for 0.5–15% of cases (Madden et al., 2002; Sanyelbhaa Talaat et al., 2009; Gohari et al., 2019), with a prevalence of approximately 0.23% in at-risk children (Rance et al., 1999); however, the diverse etiologies of AN are only beginning to be characterized. Genetic factors and the effects of a wide range of other etiologies (anoxia, certain infectious diseases, and hyperbilirubinemia) are estimated to account for 42 and 10% of all cases of AN, respectively, and the remaining 48% of cases lack a defined etiology (Starr et al., 2000, 2008). All forms of AN may be present in isolation (non-syndromic AN) or with multisystem involvement, including peripheral and/or optic neuropathies as well as various central nervous system (CNS) disorders (syndromic AN) (Santarelli, 2010).

Due to the distinct clinical and pathological features of non-syndromic AN (NSAN), its diagnosis mainly relies on audiological measures and electrophysiological tests along with ancillary methods, such as OAEs, acoustic immittance, temporal bone high-resolution computed tomography (HRCT) and magnetic resonance imaging (MRI). Pure-tone audiometry enables the degree, type, and configuration of HL to be identified. In addition to revealing impaired speech perception that is out of proportion to HL, electrophysiological tests [including ABRs and electrocochleography (ECochG)] and OAEs can assist in identifying the sites of the lesions along the auditory pathway since dysfunction of the auditory nerve, IHCs and/or IHC ribbon synapses [absent/abnormal ARs, ABRs, and action potential (AP) on ECochG] is accompanied by preserved OHC measurements (OAEs and/or CMs) (Santarelli, 2010). Furthermore, HRCT and MRI approaches have become effective methods for identifying possible structural or inflammatory abnormalities of the auditory nerve and auditory pathway that cause HL (Sharma et al., 2018). Therefore, when diagnosing AN, both imaging modalities are powerful methods for ruling out any organic lesions.

Temperature-sensitive auditory neuropathy (TSAN) is a very rare form of NSAN with distinct phenotypes. The most striking feature of TSAN is that patients present with transient HL after a rise in the core body temperature. This disorder was first described by Gorga et al. (1995) in a child with recurrent but completely reversible HL during febrile conditions. Clinical examinations showed that poor speech understanding emerged earlier than pure-tone HL and resolved more slowly than the loss of auditory function. Tests of cochlear function, including OAEs and ECochG, were normal; however, all the tests related to central connections within the brainstem, such as ARs, ABRs, and mechanically evoked trigeminofacial reflexes, were abnormal regardless of whether auditory function was normal or impaired at the time. Moreover, the middle-latency responses and auditory late potentials appeared normal. The authors predicted that the site of the lesion was the brainstem, rather than the peripheral nervous system or higher levels in the auditory pathway. Three years later, Starr et al. (1998) identified three children—two siblings and an unrelated child—who carried similar phenotypes, as confirmed by tests performed in both febrile and afebrile states. Two of the three children may have had a summating potential (SP), the existence of which was not examined in the first reported patient. Furthermore, all three patients had mild HL either at all frequencies or specifically at low frequencies when afebrile. In addition, one patient showed abnormal median sensory nerve conduction. The authors proposed that the patients’ transient deafness resulted from a demyelinating disorder of the auditory nerve and concluded that the reported cases represented an atypical form of AN, which was termed TSAN. Examples of both sporadic and familial TSAN have been described (Cianfrone et al., 2006; Varga et al., 2006; Madanoglu and Derinsu, 2007; Marlin et al., 2010; Dimitrijevic et al., 2011; Matsunaga et al., 2012; Wynne et al., 2013; Kaga, 2016; Zhang et al., 2016). Although fever-associated HL is reversible and OAEs are always normal, all reported cases have two abnormal auditory findings in common: ABRs and ARs are abnormal during both febrile and afebrile episodes. Further research revealed that variants of the *OTOF* gene are the main cause of this disease (Varga et al., 2006; Romanos et al., 2009; Marlin et al., 2010; Wang et al., 2010; Matsunaga et al., 2012; Zhang et al., 2016).

The pathogenic mechanism of TSAN is only beginning to be understood. To date, only one study performed by Strenzke et al. (2016) successfully built an *Otof*^*I*515*T/I*515*T*^ mutant mouse model, which exhibited a similar severity of HL to that in humans. Further research revealed that impaired exocytosis at hair cell ribbon synapses due to abnormal synaptic vesicles caused by Ile515Thr-otoferlin was the main pathogenic mechanism of TSAN. Notably, the mutant mice failed to show more severe hearing loss at high body temperatures, unlike the patients. The authors concluded that the lack of a human RXR motif (20 amino acids), which is thermally sensitive in mice, was the cause of inconsistent phenotypes between mutant mice and TSAN patients. However, TSAN patients are rare, the relationship between the severity of phenotypes and variants of the *OTOF* gene is still unclear, and clinical tests have never detected any demonstrable structural lesion or underlying mechanism to explain this uncommon type of HL.

Herein, we describe a TSAN-afflicted family with a distinctive pattern of phenotypic and genetic features. Four siblings, including a pair of identical twins, were examined in our clinic, and diagnosed with TSAN. Using next-generation sequencing (NGS) technology, we identified two compound heterozygous variants of the *OTOF* gene that are associated with TSAN. These data reveal genotype–phenotype correlations in TSAN, providing an improved understanding of the diagnostic features and natural history of this disease.

## Materials and Methods

### Subjects

**FIGURE 1:**
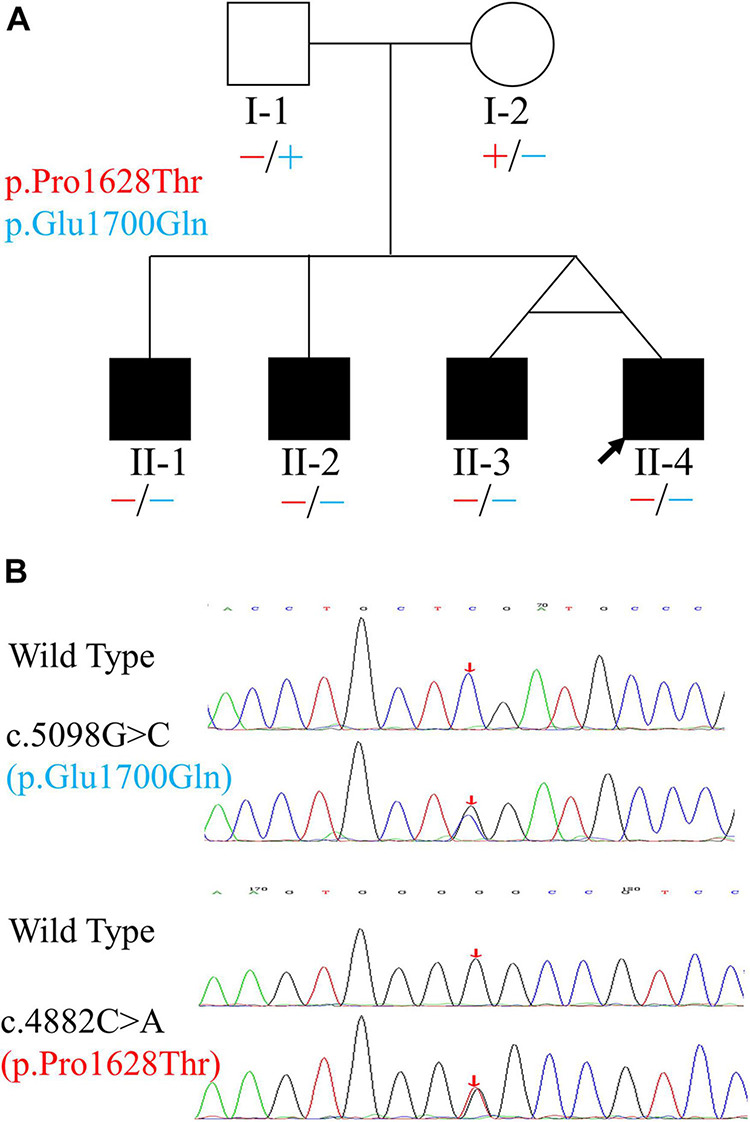
Pedigree and variants of the *OTOF* gene found in the family. **(A)** Pedigree and distribution of *OTOF* variants. Filled symbols represent affected individuals. Circle indicates female and squares indicate males. The two variants are shown in different colors. “–” and “+” represent variant and wild-type *OTOF*, respectively. **(B)** Electropherograms showing the two heterozygous *OTOF* variants (in the reverse direction). The positions of variants are indicated by red arrows.

In 2016, a 13-year-old boy with normal-hearing parents visited our clinic. The boy complained of hearing difficulty and poor speech discrimination only when his body temperature was high due to influenza or other causes. His family history revealed that his three brothers (including a twin brother) had similar complaints. Six individuals from this family were enrolled in the study (Figure 1). Detailed medical histories of the four siblings were obtained, and otologic and hearing evaluations of the four patients were performed during both afebrile and febrile states. Their parents were enrolled for the genetics portion only because neither parent complained of fluctuating HL when their body temperature varied. Vestibular function was not evaluated. Body temperature was measured at the axilla using a clinical mercury thermometer (Yuwell, Nanjing, China). Two hundred unrelated, ethnicity- and age-matched individuals (100 males and 100 females) with normal hearing were recruited as normal controls to rule out genetic polymorphisms.

The study was approved by the institutional ethics review board, including committees of medical ethics at the Nanfang Hospital, the Chinese PLA General Hospital, and the Lanzhou University Second Hospital. After written informed consent was obtained from all subjects included in the study or their guardians, genomic DNA (gDNA) was collected from the peripheral blood of all participants using standard procedures.

### Auditory Function Examination

All hearing evaluations, including pure-tone and speech audiometry, acoustic immittance, ABRs, ECochG, and distortion product otoacoustic emissions (DPOAEs), were performed according to standard clinical methods in a magnetically shielded and sound-attenuated room in the Department of Otolaryngology, Head and Neck Surgery, Nanfang Hospital, Southern Medical University, Guangzhou, China. Furthermore, imaging examinations, including thin-slice temporal bone HRCT scans and MRIs, were performed when the patients were febrile and afebrile.

#### Audiometric and Acoustic Immittance Tests

Air- and bone-conduction pure-tone audiometry was performed at octave frequency intervals in the range of 0.125–8 kHz for both ears using a GSI AudioStar Pro audiometer (Grason-Stadler Inc., Eden Prairie, MN, United States). The pure-tone average over four frequencies (0.5, 1, 2, and 4 kHz) from the better-hearing ear was used for data analysis. The severity of HL was graded as normal (0–25 dB HL), mild (26–40 dB HL), moderate (41–55 dB HL), moderately severe (56–70 dB HL), severe (71–90 dB HL), or profound (>90 dB HL) (Schlauch and Nelson, 2015). Speech audiometry measures included the speech reception threshold (SRT) and monosyllabic speech discrimination score (SDS) at 40 dB SL or less above the SRT or at the maximum level (100 dB HL) of the audiometer, depending on the patient’s hearing threshold and the highest comfortable level, especially during periods of elevated thresholds. Mandarin speech test materials (MSTM) were used to evaluate speech recognition under quiet conditions. Speech recognition tests were conducted in noise using a 10 dB signal-to-noise ratio at the baseline test. Speech testing in noise was not evaluated upon follow-up testing due to patient reports of discomfort from the noise levels used. Acoustic immittance measurements (including tympanometry and AR) were performed using an Interacoustics AT235h Impedance Audiometer (Middelfart, Denmark). The outcomes of a tympanogram with a 226 Hz probe tone were categorized as either type A, B, or C and accompanied by a cutoff negative pressure for type C at -100 daPa, as defined in the Jerger classification system (Jerger, 1970). ARs were measured ipsilaterally and contralaterally to the stimulated ear at frequencies of 0.5, 1, 2, and 4 kHz.

#### Electrophysiological Measures

Auditory brainstem responses and ECochG signals were recorded using the IHS SmartEP system (Intelligent Hearing Systems, Miami, FL, United States) with ER-3A insert earphones (Etymotic Research, Elk Grove Village, IL, United States). Click stimuli with a duration of 0.1 ms, which were separated into condensation (C) and rarefaction (R) clicks, were monaurally presented at a repetition rate of 19.3 clicks/s for ABRs and 7.1 clicks/s for ECochG. Furthermore, CMs were measured during the ABRs by examining responses to opposite-polarity stimuli, whereas ECochG was used to examine SP and AP.

Auditory brainstem responses were differentially recorded using alternating polarities, and 1,024 sweeps were averaged and analyzed using a 12-ms epoch (time window) with filtering at 100–1,000 Hz. To better evaluate and monitor the processes of auditory function recovery, stimuli were presented at the maximum level (100 dB nHL) and reduced in 10-dB steps until there was no response. For every condition, at least two replication trials were performed. ABRs were interpreted as having a CM when (1) there was a short latency (approximately 0.8 ms) or no latency shift was observed with variations in the stimulus level (Shi et al., 2012), (2) the response appeared before the normal ABR waveform, (3) the phases were inverted upon the reversal of stimulus polarities (rarefaction and condensation), and (4) clamping of the acoustic conduction tube eliminated the responses. When these criteria for CMs were met, the CM amplitude was enhanced by subtracting the average separate responses to C and R stimuli (C - R) instead of adding the traces (C + R), which resulted in the elimination of CMs.

Tympanic ECochG was performed with a wick electrode (Lilly, Miami, FL, United States) placed against the unanesthetized tympanic membrane (TM) under direct microscopic visualization. To obtain better performance, the clicks used as acoustic stimuli were also delivered at different intensity levels decreasing in 10-dB steps from a maximum intensity of 100 dB nHL to allow visual detection of the AP and SP. For the AP and SP recordings, 512 sweeps with separate opposite click polarities (C and R) as well as alternating polarities (within a single test run) were averaged using an epoch of 5 ms (including 1 ms prestimulus time) and a filter setting of 100–1,500 Hz. The AP and SP amplitudes and the SP/AP amplitude ratio were analyzed accordingly regarding the amplitude recorded. The SP/AP amplitude ratio was defined as enhanced when it was ≥0.4, in accordance with a proposed classification (Ohashi et al., 2009).

#### Distortion Product Otoacoustic Emission Test

Distortion product otoacoustic emissions were tested using a GSI Audera system (Grason-Stadler Inc., Eden Prairie, MN, United States). DPOAEs were evaluated in quarter-octave bands from 500–8,000 Hz using a frequency (F) ratio F2/F1 = 1.22 with levels (L2 and L1) of 65- and 55-dB SPL. When emissions were present at 60% or more of the test frequencies, DPOAE was defined as present; otherwise, it was defined as absent.

#### Test Frequency

**FIGURE 2:**
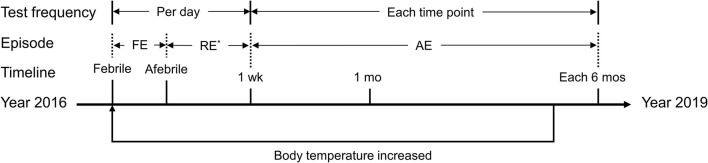
Timeline of the episodes and tests described in the present study. FE, febrile episode; RE, recovery episode; AE, afebrile episode; wk, week; mo, month. *Not available in some days during this period. The axis indicates the timeline from the year 2016 to the year 2019. The phrase “body temperature increased” indicates a round of hearing fluctuation due to a febrile episode. During the follow-up period, the proband experienced three febrile episodes, whereas each of the other three patients experienced only one. Tests were completed at each time point during each febrile episode.

Patients enrolled in this study were followed up for 3 years at the local hospital. Hearing tests were performed daily during febrile episodes and then repeated every day within the first week (when possible) after each febrile event as well as one month later and every 6 months thereafter (Figure 2). Otitis and other causes of HL were first excluded during these febrile episodes by physical and imaging examinations.

#### Analysis of Clinical Features

To better interpret clinical presentations and diagnostic evaluations, we divided the course of each febrile event into three separate periods, i.e., the febrile episode, the recovery episode and the afebrile episode, referring to the day(s) when the patient had a fever, the first 6 days after body temperature recovery, and the period beginning 1 week after body temperature recovery, respectively (Figure 2). The results were calculated and plotted using the Origin 8.5 package (OriginLab Corp., Northampton, MA, United States). Data are presented as the mean ± SEM (standard error of the mean).

### Genetic Analysis

#### Determination of Twin Zygosity

To confirm whether the twin brothers were identical or fraternal, the AGCU 17 + 1 STR Kit (AGCU ScienTech Inc., Wuxi, China), which is composed of 17 autosomal unlinked loci and the sex-determining marker amelogenin, was used to determine the twin zygosity. The autosomal loci included D3S1358, D13S317, D7S820, D16S539, Penta E, TPOX, TH01, D2S1338, CSF1PO, D19S433, vWA, D5S818, FGA, D6S1043, D8S1179, D21S11, and D18S51. Polymerase chain reaction (PCR) amplification, sample preparation, and electrophoresis were performed in accordance with the manufacturers’ instructions (Fan et al., 2012). Briefly, gDNA (0.5–2.0 ng) from the two twin brothers was added to the reaction mix along with HotStart C-Taq DNA polymerase and 17 + 1 fluorescently labeled primers to give a final volume of 10 μL for PCR (conditions not shown). The targeted products were analyzed using an ABI 3130XL Genetic Analyzer (Applied Biosystems, Foster City, CA, United States). Genotypes were validated by comparing the sizes of the unknown fragments to the allelic ladders provided by the kit.

#### Targeted Next-Generation Sequencing

After the common deafness genes (*GJB2*, *SLC26A4*, and *MT-RNR1*) had been excluded by direct sequencing (Du et al., 2014), NGS technology was applied to identify the causative gene in this family.

For targeted capture and massively parallel sequencing (MPS), the qualified gDNA of the proband (II-4) was randomly sheared using the Covaris S2 Focused Ultrasonicator (Covaris, Massachusetts, MA, United States) to an average fragment size of 350–400 bp. The fragments were then end-repaired, ligated to adapters, and analyzed using an Agilent 2100 Bioanalyzer. All exons and flanking intronic regions of 159 deafness-related nuclear genes, 6 deafness-related mitochondrial regions, and 3 miRNAs (Supplementary Tables 1–3) were captured using a GenCap kit (MyGenostics, Beijing, China). The captured sequences were analyzed by high-throughput sequencing using a NextSeq 500 next-generation sequencer (Illumina Inc., San Diego, CA, United States).

To identify modifier genes for the phenotypic manifestation of TSAN-associated *OTOF* variants, we performed whole-exome sequencing (WES) of DNA from the proband (II-4). For WES confirmation, fragment libraries were prepared using the Nextera Rapid Capture kit (Illumina). The gDNA fragments were end-repaired and purified in accordance with the manufacturer’s protocol. The Nimblegen SeqCap EZ Exome v3.0 (64 Mb) Kit (Roche, Madison, WI, United States) was used to capture exons after the manufacturer’s protocols, and these exons were then sequenced on the HiSeq XTen PE150 platform (Illumina).

#### Bioinformatics Analysis

Next-generation sequencing data from the two procedures were analyzed using an in-house bioinformatics pipeline, as we previously described (Yuan et al., 2020). Briefly, qualified reads were obtained after low-quality data were filtered out from the raw data using the Cutadapt program (Martin, 2011) and were then mapped to the human reference genome (GRCh37/hg19) using the program Burrows-Wheeler Aligner (BWA).^1^ Subsequently, the Genome Analysis Toolkit (GATK) program was used to call single-nucleotide variants (SNVs) and insertions or deletions (indels). The SNVs and indels were annotated using public databases (including the 1000 Genomes, gnomAD/ExAC, ClinVar, HGMD, and ClinGen databases and the Deafness Variation Database) and in-house databases according to the ACMG/AMP guidelines for genetic HL (Oza et al., 2018).

#### Variant Confirmation and Screening

The suspected candidate variants were confirmed by Sanger sequencing, and the responsible variants were identified based on co-segregation analysis with the TSAN phenotype among the family members. The primer sequences and PCR conditions are available upon request. Finally, the detected variants were screened in the control group to discard polymorphisms and explore the allele frequencies according to recessive inheritance patterns.

#### Evolutionary Conservation and Molecular Model Analyses

Evolutionary conservation was evaluated across 11 organisms using Clustal X 2.1 (Larkin et al., 2007). To analyze the effects of the two identified *OTOF* variants, the three-dimensional (3D) structures of otoferlin and its p.Pro1628Thr and p.Glu1700Gln mutants were modeled using AlphaFold (Jumper et al., 2021). The molecular data obtained by homology modeling were represented using the PyMOL 2.5 molecular graphics system.^2^

## Results

### Clinical Data and Audiological Findings

**FIGURE 3:**
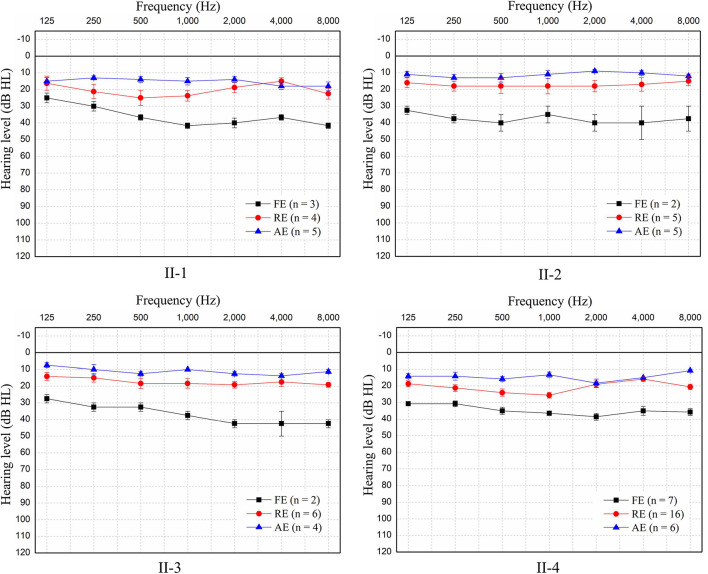
Pure-tone audiometry of the four patients during the three episodes. Since no air-bone gap was found, only pure-tone air-conducted thresholds for the better ear for each patient are shown. Patients experienced mild sensorineural hearing loss during each febrile episode (FE), but hearing returned to normal in the recovery episode (RE) and remained normal in the afebrile episode (AE). The hearing threshold at each frequency is shown as the mean ± SEM of the air-conduction threshold. N: number of test events.

Four siblings were clinically diagnosed with non-syndromic TSAN based on auditory evaluations, medical histories, and physical examinations. The relationship among the siblings is shown in Figure 1. They complained of hearing impairment and a poor ability to discriminate speech during each febrile episode. This phenotype of TSAN was associated with communication difficulties secondary to poor auditory function during febrile episodes, and speech recognition abilities recovered immediately when the core body temperature returned to normal. Although general physical examinations identified no structural or developmental abnormality in any of the four siblings, audiometry results showed that their SDSs improved more rapidly than other test results, such as those for ECochG signals and ABRs. Interestingly, pure-tone thresholds improved from mild HL during the febrile condition to normal levels during the afebrile periods (Figure 3 and Table 1), while CMs were present during all test conditions (Table 1). Moreover, the follow-up audiometry assessments, except for the continued absence of ARs, showed improvement to normal levels during afebrile episodes. Importantly, HRCT and MRI scans failed to show any structural or inflammatory abnormalities or abnormal contrast enhancement of the ear (including the outer, middle, and inner ear), auditory nerve or brain, such as tympanitis, acoustic neuromas or brainstem tumors, in febrile or afebrile episodes. Notably, their unaffected parents without similar complaints had clinically normal hearing ability and were able to recognize speech.

**TABLE 1:** Overview of genotype–phenotype correlations of *OTOF* variants in the TSAN patients identified in the present study or previous studies.

	**Family 1**	**Family 2**	**Family 3**	**Family 4**	**Family 5**	**Family 6**	**Family 7**	**Family 8**
**Genotype**								
*OTOF* variants	c.1544T > C/ c.3346C > T	c.1841G > A/ c.3239G > C	c.5410_ 5412del GAG Hom	c.1621G > A Hom	c.2975_2978delAG/ c.4189C > T	c.4189C > T Hom	c.2382_2383delC/ c.1621G > A	c.5098G > C/ c.4882C > A
Otoferlin changes	p.Ile515Thr/ p.Arg1116Ter	p.Gly614Glu/ p.Arg1080Pro	p.Glu1804del Hom	p.Gly541Ser Hom	p.Gln994Valfs*7/ p.Arg1607Trp	p.Arg1607Trp Hom	p.Leu795Serfs*5/ p.Gly541Ser	p.Glu1700Gln/ p.Pro1628Thr
No. of CAV	2	1	2	2	2	2	2	0
**Phenotype**								
No. of patient(s)	2	1	3	1	1	1	1	4
Origin	United States	Brazil	Scotland	Japan	China	China	China	China
Sex	Male (1), Female (1)	Female	Male (1), Female (2)	Male	Male	Male	Male	Male
Age at onset (diagnosis)	2 (3, 2) years	–	2 (7–10) years	10 (25) years	13 months	6 years	30 months	Childhood (8–15 years)
**Febrile episode**								
Body temperature (°C)	38.1/37.8	–	>38	37.2	36.64^†^/36.5	36.9	–	38–40.2
Hearing level	Profound-mild	Severe	Profound/severe	Profound	Severe^†^/moderate	Mild	–	Mild
SDS (%)	0	–	<100	≤15	–^†^/(16–20)	88–80	–	0–20
Tympanometry	A	–	–	–	A	A	A	A × 3/C ×1
AR	Absent	–	–	–	Absent	Absent	Absent	Absent
OAEs	Present	–	Present	Present	Present	Present	Present	Present × 3/Absent × 1
ABR	Absent	–	Abnormal	Absent	Absent	Absent	Absent	Absent
CM	Present	–	–	Present	Present	Present	Present	Present
ECochG	Absent	–	–	Abnormal	–	–	–	Absent
**Afebrile episode**								
Body temperature (°C)	Normal	–	–	36.8	(36.13–36.32)^†^/36.3	36.6	–	36.3–37.3
Hearing level	Mild in low frequencies	Mild	Normal/mild	Mild	Moderately severe^†^/mild	Moderate^†^/normal	Moderate	Normal
SDS (%)	88–100	–	≤80	≤80	–^†^/16	96	93–98 (after CI)	88–100
Tympanometry	A	–	–	–	A	A	A	A
AR	Absent	–	–	–	Absent	Absent	Absent	Absent
OAEs	Present	Present	Present	Present	Present	Present	Present	Present
ABR	Abnormal	Abnormal	Abnormal	Absent	Absent	Absent	Absent	Normal
CM	Present	–	–	Present	Present	Present	Present	Present
ECochG	SP (possible)	–	–	SP (definite)	Abnormal	–	–	Normal
Auditory rehabilitation	–	–	Hearing aids	–	Improved with age	Improved with age	Cochlear implantation (CI)	No^‡^
References	Starr et al., 1998; Varga et al., 2006; Strenzke et al., 2016	Romanos et al., 2009	Marlin et al., 2010	Matsunaga et al., 2012; Kaga, 2016	Wang et al., 2010; Zhang et al., 2016	Zhang et al., 2016	Zhang et al., 2016	Present study

*^–^, undefined/untested; ^†^, before hearing improvement; ^‡^, automatically recovered. ABR, auditory brainstem response; AR, acoustic reflex; CAV, C2 domain-affecting variant of otoferlin; CM, cochlear microphonic; ECochG, electrocochleography; Hom, homozygote; OAE, otoacoustic emission; SDS, speech discrimination score.*

During the 3-year follow-up period, the proband suffered from three episodes of “poor hearing,” while his three brothers experienced one episode each. Audiological tests revealed that all four brothers had the same pattern of hearing fluctuation. Because of the different auditory phenotypes and test results for the different periods, the results from the febrile, recovery and afebrile periods are shown separately (Table 2).

**TABLE 2:** Clinical features of four brothers with temperature-sensitive auditory neuropathy.

	**II-1**	**II-2**	**II-3**	**II-4**
Sex	Male	Male	Male	Male
Age^†^ (years)	16	14	13	13
***Febrile episode***				
*N*	3	2	2	7
Temperature	37.9 ± 0.1	37.7 ± 0.2	38 ± 0.2	38.4 ± 0.3
Hearing level	Mild	Mild	Mild	Mild
Average SDS (%, L/R)	4/12	10/15	13/5	5/4
Tympanogram	A	A	A	C
AR	Abs	Abs	Abs	Abs
ABR	Abs	Abs	Abs	Abs
CM	Pres	Pres	Pres	Pres
ECochG	Abs	Abs	Abs	Abs
DPOAE	Pres	Pres	Pres	Abs^‡^
***Recovery episode***				
*N*	4	5	6	16
Temperature	36.6 ± 0.1	36.2 ± 0.1	36.5 ± 0.1	36.5 ± 0.1
Hearing level	Normal	Normal	Normal	Normal
Average SDS (%, L/R)	98/97	97/96	98/99	97/97
Tympanogram	A	A	A	C × 6/A × 10
AR	Abs	Abs	Abs	Abs
ABR	Abs × 2/Abn × 2	Abs × 3/Abn × 2	Abs × 4/Abn × 2	Abs × 6/Abn × 10
CM	Pres	Pres	Pres	Pres
ECochG	Abs × 2/Abn × 2	Abs × 3/Abn × 2	Abs × 4/Abn × 2	Abs × 6/Abn × 10
DPOAE	Pres	Pres	Pres	Abs^‡^ × 6/Pres × 10
***Afebrile episode***				
*N*	5	5	4	6
Temperature	36.3 ± 0.1	36.2 ± 0.1	36.4 ± 0.1	36.5 ± 0.1
Hearing level	Normal	Normal	Normal	Normal
Average SDS (%, L/R)	99/98	98/97	98/99	98/99
Tympanogram	A	A	A	A
AR	Abs	Abs	Abs	Abs
ABR	Normal	Normal	Normal	Normal
CM	Pres	Pres	Pres	Pres
ECochG	Normal	Normal	Normal	Normal
DPOAE	Pres	Pres	Pres	Pres

*^†^At the time of enrollment in the study.*

*^‡^Absent DPOAEs may have been due to abnormal middle ear function.*

*N, number of test events (results are not available for several days during recovery episodes); ABR, auditory brainstem response; AR, acoustic reflex; CM, cochlear microphonic; DPOAE, distortion product otoacoustic emission; ECochG, electrocochleography; SDS, speech discrimination score; L/R, left/right ear; Abn, abnormal (detectable with an elevated threshold and/or prolonged latency for ABRs or an enlarged SP/AP amplitude ratio for ECochG); Abs, absent (no detectable wave or response); Pres, present.*

#### Febrile Manifestations

**FIGURE 4:**
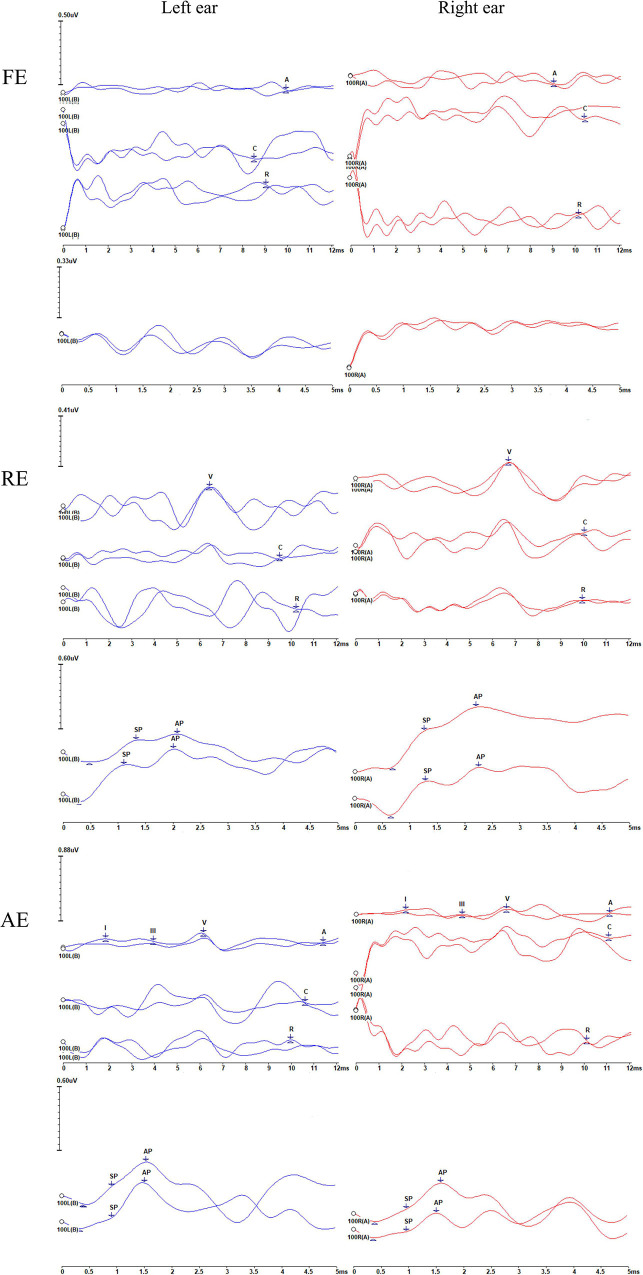
Recordings of the auditory brainstem responses (ABRs), cochlear microphonics (CMs), and electrocochleography (ECochG) waveforms of the proband (II-4) during the three episodes. FE, febrile episode; RE, recovery episode; AE, afebrile episode. For each episode, the upper and lower panels represent the ABRs and CMs and ECochG signals, respectively. The number 100 indicates the click stimulus level. “A” indicates ABRs, and “C” and “R” represent the condensation and rarefaction polarity click responses, respectively. CMs can be identified according to the inverted phases of C and R responses. Waves I, III, and V are marked. The changes in ABRs and CMs differ. ABRs were gradually restored, i.e., absent (FE), then abnormal (with an elevated threshold and prolonged latency) (RE), and finally, almost normal (AE). However, CMs were persistently present, although some were of low amplitude. AP and SP represent the action potential and summating potential, respectively. ECochG signals were absent under febrile conditions (FE). Notably, the results appear show neuroelectric activity and possibly the CMs rather than ECochG. During the RE, the SP/AP amplitude ratios were >0.4. During the AE, the SP/AP amplitude ratios were <0.4, indicating a recovery of auditory function.

The pure-tone thresholds were not consistent with those of SDSs or the electrophysiological examinations of the cochlea. During febrile episodes, the pure-tone thresholds of the four patients indicated bilateral sensorineural mild HL (Figure 3), but the average SDSs were very low, varying from 4 to 15% under quiet conditions (Table 2). The tympanograms were type C for the proband but type A for the other patients, and ARs (both the ipsilateral and contralateral sides) were consistently absent for both ears from 500 to 4,000 Hz (Table 2). Bilateral DPOAEs were absent in the proband but present in the other three brothers (Table 2). The neural components of the ABR and the ECochG (AP) were unanimously absent during febrile episodes (Figure 4). In contrast, CMs were observed bilaterally in response to a stimulus presented at 100 dB nHL (Table 2 and Figure 4).

#### Recovery Manifestations

During the recovery period, auditory function improved, but it did so at variable rates. Pure-tone audiometry and SDS returned to normal quickly, whereas the ABRs and ECochG signals progressed from absent to abnormal before recovering to normal (Table 2 and Figure 4). The SDSs exhibited faster recovery than the metrics of the other audiological tests. Although the DPOAEs and tympanograms of the proband transitioned to present and type A, respectively, those of his brothers remained normal (present DPOAEs and type A tympanograms) (Table 2). The ARs and CMs of all four patients were absent and present, respectively.

#### Afebrile Manifestations

During afebrile episodes, audiological tests showed consistent normal results in pure-tone audiometry, SDSs, DPOAEs, ABR, and ECochG along with absent ARs (Table 2 and Figures 3, 4). These results indicated that the auditory function of all patients recovered despite the continued absence of ARs.

### Genetics Analysis

#### Determination of Twin Zygosity

All 18 loci investigated with the AGCU 17 + 1 STR Kit showed a consistent allelic pattern; therefore, the twin boys were concluded to be monozygotic (identical) (Supplementary Table 4). The 18 investigated loci provided a matching probability high enough to assume that the results were accurate and reliable (Fan et al., 2012).

#### Targeted Next-Generation Sequencing and Data Analysis

To identify the cause of HL in the family, we first performed targeted NGS analysis to identify the possible variant(s) present in the proband. For MPS, the coverage and average read depth of the targeted regions were 98.26% and 350-fold, respectively. Of the 496 identified variants (Supplementary Table 5), seven candidate variants remained after filtering (Supplementary Table 6). For WES, the data mapped to the targeted region had a mean depth of 194.57-fold, and the coverage of the targeted bases was 99.19% at a depth of 4×, 98.11% at a depth of 10×, and 96.15% at a depth of 20×. A total of 64,940 variants remained after we filtered out those with allele frequencies greater than 5% in the 1000 Genomes, gnomAD/ExAC and in-house databases. For further analysis, we focused only on variants in splicing and coding regions. After completing this filtering process, we identified 1,381 variants (Supplementary Table 7). Finally, seven variants remained after referring to the variation databases [ClinVar, HGMD and Online Mendelian Inheritance in Man (OMIM)] and literature (Supplementary Table 8). Under the autosomal recessive mode of inheritance, two variants in two genes (*MHN14* and *OTOF*) from both sequencing processes were selected for further analysis. Notably, we failed to identify any known gene carrying a compound heterozygous or homozygous variant using WES.

#### Variant Validation and Analysis

**FIGURE 5:**
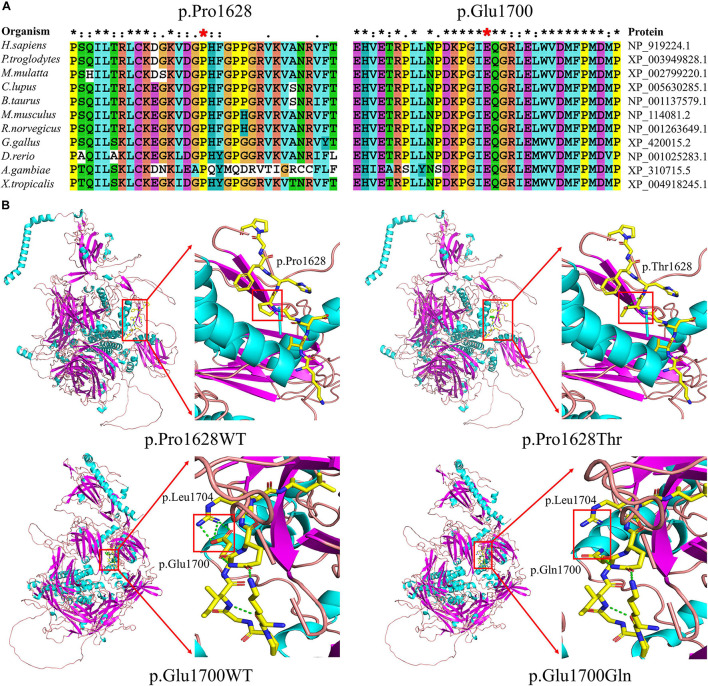
Results of evolutionary conservation and molecular model analyses. **(A)** Amino acid conservation analysis. Protein alignment shows that the Pro1628 **(left)** and Glu1700 **(right)** residues of otoferlin are highly conserved across 11 organisms. The two positions of Pro1628 and Glu1700 are indicated by red stars. **(B)** Molecular models of wild-type (WT) **(left column)** and missense variants **(right column)** in otoferlin. The variant p.Pro1628Thr was found to perturb an amino acid chain and generate an extra hydrogen bond within the mutant p.Thr1628 **(upper row)**. The variant p.Glu1700Gln likely perturbs an amino acid side chain and has lost the hydrogen bonds between p.Glu1700 and p.Leu1704 **(lower row)**.

Using PCR-Sanger sequencing technology, variants in both *MYH14* (c.1133C > T and c.1301A > G, RefSeq: NM_001145809.1) and *OTOF* (c.5098G > C and c.4882C > A, RefSeq: NM_194248.2) genes were validated. The results revealed that only the two variants of the *OTOF* gene completely co-segregated with deafness in this family. In the three cases (with the identical twin brothers regarded as representing one case) involving subjects who were TSAN patients, the subjects were compound heterozygotes for the two variants, and both parents were unaffected and heterozygous (Figure 1). The c.5098G > C variant, which occurred in exon 40 and was inherited from the unaffected mother (I-2), resulted in a glutamic acid-to-glutamine substitution at position 1,700 and has been previously reported as a pathogenic allele (Chiu et al., 2010; Chen et al., 2018; Qiu et al., 2019; Wu et al., 2019). The other variant, c.4882C > A, which was located in exon 39 and passed on from the clinically normal father (I-1), resulted in a single amino acid change from a proline to a threonine at position 1,628 in otoferlin. This variant was first identified in this study and has a high Rare Exome Variant Ensemble Learner (REVEL) score (0.742) and a very low allele frequency in the public database ExAC (8.67 × 10^–6^). Furthermore, both variants were located at conserved amino acid positions of the otoferlin protein (Figure 5A). Based on these results, the phenotypes of the family, and the ACMG/AMP rules for recessive HL, the novel variant (c.4882C > A) was classified as pathogenic according to the standards of PS4, PM2, PM3, PP1_Strong, PP3 and PP4 (Oza et al., 2018). The patient’s phenotypes and the detected variants have been deposited into ClinVar under accession numbers SCV001787151 and SCV001787152.

#### Variant Screening

Both variants identified in this study were absent in 200 normal-hearing controls matched for Chinese ethnicity, suggesting that *OTOF* was the causative gene of the autosomal recessive trait in this family.

#### Results of Evolutionary Conservation and Molecular Modeling

The positions of both Proline1628 and Glutamic acid1700 in otoferlin are conserved across 11 organisms (Figure 5A). 3D structural analysis revealed that both variants were likely to perturb amino acid (side) chains (Figure 5B).

## Discussion

In the mammalian inner ear, hair cells (HCs) transform mechanical vibrations into electrical signals (Wang et al., 2017; He et al., 2019; Liu Y. et al., 2019; Qi et al., 2019, 2020; Jiang et al., 2020; Zhou et al., 2020), and spiral ganglion neurons (SGNs) function as electric signal-transduction cells (Sun et al., 2016; Liu et al., 2018, 2021; Guo et al., 2019, 2020, 2021; Zhao et al., 2019). While supporting cells function as potential resources to regenerate HCs after damage (Lu et al., 2017; Zhang et al., 2017; Tan et al., 2019; Zhang S. et al., 2019, 2020; Zhang Y. et al., 2020; Chen et al., 2021). Sensorineural hearing loss has many etiologies, varying from genetic defects (Zhu et al., 2018; Fang et al., 2019; Yang et al., 2019; Qian et al., 2020; Cheng et al., 2021; Fu et al., 2021a; Lv et al., 2021; Zhang et al., 2021) to environmental factors, including aging (Zhang et al., 2017; Fu et al., 2018; Li H. et al., 2018; He et al., 2020, 2021), noise and ototoxic drugs (Liu et al., 2016; He et al., 2017; Li A. et al., 2018; Zhang Y. et al., 2019; Gao et al., 2020; Zhong et al., 2020; Fu et al., 2021b). TSAN is a rare phenotype with high clinical heterogeneity, manifesting as transient bilateral HL due to an increase in the core body temperature. The transient, conditional nature of this HL makes diagnosis challenging. In the present study, the diagnosis of AN was based on normal CM responses and abnormal ABRs during febrile episodes, although the tympanometry and AR measurement results were abnormal in one of four cases at the beginning of febrile episodes. However, further audiometric evaluations showed that the patients’ behavioral thresholds, ABRs, and ECochG signals recovered to normal during afebrile episodes. Unlike other patients with TSAN or other forms of AN, the ECochG pattern in our patients during afebrile episodes was reversible, suggesting that auditory function almost completely recovered, although ARs continued to be absent. Therefore, certain exceptions to the usual features of AN often need to be considered when making a diagnosis, as 40% of affected individuals have abnormal rather than absent ABRs, ARs are sometimes present at elevated thresholds rather than being absent, and OAEs are absent in approximately 30% of AN patients upon retesting (Starr et al., 2001, 2008). In the present study, the proband’s abnormal DPOAEs with a type C tympanogram and absent ARs during the febrile state were very likely attributable to dysfunction of the middle ear rather than OHC and not related to TSAN, although no abnormality was detected in physical or neuroimaging examinations. More notably, however, negative middle ear pressure should result in a retracted TM, which may or may not be observable upon physical examination, depending on the experience of the person conducting the examination, the amount of negative pressure, and the use of a standard otoscope versus microscopic examination of the TM. Furthermore, imaging studies will not show a retracted TM as an isolated finding. In contrast, his twin brother (II-3) showed consistent normal DPOAEs and a type A tympanogram, regardless of the body temperature, although they shared the same genotype. However, the changes in CMs and ABRs during each febrile event in the twins were the same, indicating that both tests are more reliable than others when diagnosing AN, especially when DPOAEs and tympanograms differ among patients in the same family. Consequently, present CMs with absent ABRs are recommended to diagnose AN (Mittal et al., 2012). Therefore, patients in this study were diagnosed with AN when febrile despite the recovery of their auditory function after fever.

**FIGURE 6:**
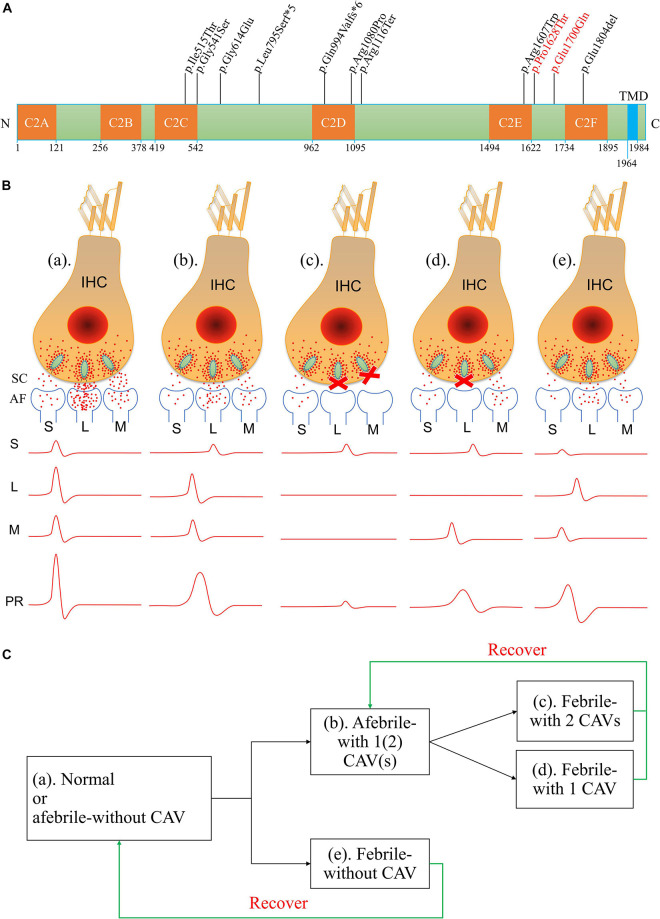
Amino acid variants in otoferlin and possible mechanisms of otoferlin-dependent exocytosis in the inner hair cell (IHC) ribbon synapses of different temperature-sensitive auditory neuropathy (TSAN) patients. **(A)** Locations of otoferlin protein variants identified in TSAN patients. The numbers indicate the domain boundaries of otoferlin. The orange boxes indicate calcium-binding domains C2A through C2F, whereas the light-blue box indicates the transmembrane domain (TMD). Eleven variants have been found to be associated with TSAN, of which three variants (p.Gly614Glu, p.Glu1700Gln and p.Pro1628Thr) do not affect any otoferlin C2 domain. Two variants identified in this study (p.Glu1700Gln and p.Pro1628Thr) are shown in red. **(B)** Abnormal neural representation of sounds due to the dysfunction of synaptic transmission caused by *OTOF* variants in normal-hearing and TSAN patients. **(a)** Normal auditory function with normal synaptic transmission between IHCs and spiral ganglion neurons (SGNs). **(b)** TSAN patients with one or two C2 domain-affecting variants (CAVs) showed persistent partial dysfunction of all IHC–SGN synapses even when afebrile. **(c)** TSAN patients with two CAVs in a febrile state. Synaptic transmitter release was transiently blocked in almost all IHC–SGN synapses in addition to the more seriously affected remaining impaired synapses than those in panel **(b)**. **(d)** TSAN patients with only one CAV in a febrile state. A large number of IHC–SGN synapses temporarily failed to function. In addition, the remaining impaired synapses were more heavily affected in this state than in panel **(b)**. **(e)** TSAN patients with no CAVs in a febrile state. The IHC–SGN synapses were slightly affected but less severely affected than those of patients with one or two CAVs. The red dots represent glutamate (neurotransmitter), and the letters “S,” “L,” and “M” indicate small, large, and medium numbers, respectively, of IHC–SGN synapses. SC, synaptic cleft; AF, afferent fiber; PR, potential recording. **(C)** The genotype–phenotype relationships between *OTOF* variants and TSAN in patients with variable auditory function in this study and previous studies.

Although several genes are involved in AN, only mutations of the *OTOF* gene are responsible for TSAN (Del Castillo and Del Castillo, 2012). To date, five loci (including four identified genes) and six genes are known to be associated with NSAN: DFNB9 (*OTOF* gene), DFNB59 (*PJVK* gene), and *GJB2* for autosomal recessive AN; AUNA1 (*DIAPH3* gene), AUNA2 (undefined gene), *SLC17A8*, *PCDH9*, *DIAPH1*, and *TMEM43* for autosomal dominant AN; DFNX5 (*AIFM1* gene) for X-linked recessive AN; and mitochondrial 12S rRNA (T1095C) (Varga et al., 2003; Cheng et al., 2005; Wang et al., 2005; Delmaghani et al., 2006; Ruel et al., 2008; Santarelli et al., 2008; Grati et al., 2009; Schoen et al., 2010; Zong et al., 2015; Lang-Roth et al., 2017; Wu et al., 2020; Jang et al., 2021). However, variants of the *OTOF* gene have been shown to be major contributors to NSAN (Varga et al., 2006; Rodríguez-Ballesteros et al., 2008; Romanos et al., 2009; Chiu et al., 2010; Wang et al., 2010). Moreover, DNA variations in *OTOF* differ among regions and ethnic populations (Rodríguez-Ballesteros et al., 2008; Del Castillo and Del Castillo, 2012; Matsunaga et al., 2012; Almontashiri et al., 2018). Two families with familial TSAN have been shown to carry *OTOF* variants. The first family was reported by Starr et al. in 1998 (Starr et al., 1998). Varga et al. (2006) showed that in this family, the two siblings with TSAN carried a heterozygous c.1544T > C (p.Ile515Thr) variant in the *OTOF* gene (Varga et al., 2006). This variant was inherited from their unaffected father and was absent in their mother and maternal siblings, and it is known to contribute to profound prelingual HL in humans and TSAN or NSAN in mouse models (Mirghomizadeh et al., 2002; Strenzke et al., 2016; Michalski et al., 2017). Further study revealed that the other mutant allele containing c.3346C > T (p.Arg1116Ter) in the *OTOF* gene (Strenzke et al., 2016). The second familial TSAN cluster was found in a consanguineous family with three members carrying a homozygous p.Glu1804del variant (Marlin et al., 2010). Furthermore, several sporadic cases of TSAN have been reported to be caused by variants in the *OTOF* gene (Romanos et al., 2009; Matsunaga et al., 2012; Zhang et al., 2016). To date, a total of nine variants of the *OTOF* gene have been identified as associated with TSAN (Table 1 and Figure 6A). In this study, we reported a third cluster of familial TSAN, which includes three cases with two variants of the *OTOF* gene (which encodes otoferlin protein), c.5098G > C (p.Glu1700Gln) and c.4882C > A (p.Pro1628Thr). The reasons why both variants were considered part of the cause of TSAN in this family are as follows: (1) both are missense variants affecting residues that are completely preserved among different species; (2) p.Glu1700Gln has been shown to be pathogenic (Chiu et al., 2010; Chen et al., 2018; Qiu et al., 2019; Wu et al., 2019); (3) p.Pro1628Thr, which is reported here for the first time, was absent in 200 normal controls and has a very low allele frequency in a public database (8.67 × 10^–6^ in ExAC); and (4) both variants strongly co-segregated with the TSAN phenotype.

Most patients with NSAN caused by *OTOF* variants present with severe prelingual or profound deafness; however, the phenotypes of TSAN patients reported in this and previous studies did not follow this pattern. In previous studies, most TSAN patients had variable HL (normal to mild) when afebrile, and their hearing thresholds dropped to a greater degree of HL, with associated communication difficulties only during febrile episodes. In addition, patients with AN caused by *OTOF* variants tended to have more frequently affected and more severely affected DPOAEs than those with no *OTOF* variants (Kitao et al., 2019). Otoferlin, which is encoded by the *OTOF* gene, is a six-C2 domain (C2A–F) protein that also has a transmembrane domain (TMD), and its function is auditory neurotransmission at the hair cell ribbon synapse in the inner ear (Roux et al., 2006; Vogl et al., 2016; Michalski et al., 2017). C2 domains have been implicated in Ca^2+^ binding, and they play a crucial role in the function of otoferlin, which may contribute to the location specificity of most pathogenic variants of the *OTOF* gene (Del Castillo and Del Castillo, 2012). Notably, at least one of the alleles observed in these cases carries a missense variant affecting a C2 domain of otoferlin (Figure 6A). Moreover, subjects with two C2 domain-affecting variants presented with more severe phenotypes than did those with only one (Table 1). Relative to subjects in earlier studies, the three subjects in this study showed mild phenotypes, with the improvement in pure-tone thresholds from mild HL during the febrile condition to normal levels during the afebrile periods and the recovery of auditory function (except for ARs) during afebrile periods. Neither of the variants identified in this study is located in a C2 domain (both are between domains C2E and C2F); in addition, the p.Glu1700Gln variant has been shown to cause progressive mild to moderate prelingual HL, and OAEs in patients with AN carrying homozygous or compound heterozygous p.Glu1700Gln may be consistently present, consistently absent, or even present in the first test and absent later (Chiu et al., 2010; Wu et al., 2018). However, in the present study, the causes of the proband’s absent DPOAEs only when febrile are still unknown, although he has the same genetic background as his twin brother. Remarkably, we did not identify modifiers using WES technology, suggesting that the variable phenotypes associated with these genotypes may result from environmental and/or genetic factors that have not yet been identified. In addition, the temperature-sensitive phenotype of HL may also be a direct consequence of defects in normal otoferlin-mediated synaptic vesicle trafficking in the inner ear. This issue may be addressed using temperature-sensitive variants that have been reported in several human diseases, including albinism and cystic fibrosis (King et al., 1991; Sharma et al., 2001; Wang et al., 2008), and decreased activity of otoferlin due to structural changes with rising temperatures may have an additive effect on the phenotype, as verified in previous reports (Strenzke et al., 2016).

Although the precise synaptic mechanism of the action of otoferlin at hair cell ribbon synapses remains unclear, the impairment of otoferlin in temperature-sensitive synaptic neurotransmitter release has been suggested to contribute to the phenotype of TSAN (Kaga, 2016; Kindt and Sheets, 2018). Normal auditory function depends on faithful information transfer, which requires otoferlin-dependent IHC exocytosis to be indefatigable, highly efficient, and accurately synchronized (Johnson, 1980; Griesinger et al., 2005; Roux et al., 2006; Kitcher et al., 2021). Reliable and temporally precise cochlear potentials are characterized by fast rise times, short onsets, and short peak latencies (Liu W. et al., 2019; Rutherford et al., 2021). Based on the findings from the present and other studies (Table 1), the severity of the phenotype associated with TSAN due to dysfunctional neurotransmitter release appears to reflect variants that alter the C2 domains of otoferlin. Therefore, the patients in previous studies had more severe HL in the febrile and afebrile states than the patients in the present study. We suggest that the possible pathogenesis and phenotype-genotype relations of TSAN caused by the dysfunction of otoferlin-dependent exocytosis in the IHC ribbon synapses are shown in Figures 6B,C. According to this hypothesis, three different clinical phenotypes may be interpreted. First, in TSAN patients with two C2 domain-affecting variants (CAVs) who became febrile, synaptic transmitter release was transiently blocked in almost all IHC–SGN synapses, especially the more seriously affected remaining synapses. The recorded cochlear current waves were low and prolonged or, in some cases, were hardly detectable. Second, when febrile, TSAN patients with only one CAV had better hearing thresholds than those with two CAVs. Large numbers of IHC–SGN synapses temporarily failed to release vesicles, with the remaining synapses being more heavily affected than those during afebrile states. Moreover, the initiation and propagation of spikes along the SGNs tended to vary in time and intensity. The measurements revealed the occurrence of cochlear potentials of variable size and shape in this type of TSAN patient under febrile conditions. Third, as in TSAN patients with one or two CAVs, mutant otoferlin in TSAN patients with no CAV exhibited impaired neurotransmitter release at IHC–SGN synapses. However, in a febrile state, the impairment caused by mutant otoferlin was less severe in TSAN patients with no CAV than in patients with one or two CAVs. As a result, synaptic transmission was inhibited for a very short period in the febrile state and rapidly increased after the body temperature decreased. The cochlear potentials, i.e., ABRs and ECochG signals, improved from no response to almost normal after the body temperature dropped. Importantly, the small magnitude of the effect on synaptic transmission may be the main reason why fever-related auditory dysfunction is almost completely reversible (with continued absent ARs) in TSAN patients with no CAV. Although the real molecular mechanisms are only beginning to be understood, otoferlin-induced defective synaptic transmission at the IHC ribbon synapse is one of the most important underlying causes of TSAN.

## Conclusion

In this study, we described the detailed courses of disease in four male siblings from a non-consanguineous Chinese TSAN family who complained of communication difficulties when febrile, and we explored the genetic etiology of their condition using NGS technology. We investigated the detailed clinical progression of autosomal recessive TSAN in four siblings with two *OTOF* variants that did not affect any C2 domain of otoferlin, and we also identified a unique characterization of normal ABRs in afebrile episodes that was different from previously reported cases. Moreover, the presence of CMs with absent (or markedly abnormal) ABRs is a reliable diagnostic criterion for AN. These observations enrich the current knowledge of the phenotypes and genotypes of TSAN and may lay a foundation for further studies of its pathogenesis.

## Data Availability Statement

The datasets presented in this study can be found in online repositories. The names of the repository/repositories and accession number(s) can be found below: the ClinVar repository (https://www.ncbi.nlm.nih.gov/clinvar/), accession numbers: SCV001787151 and SCV001787152.

## Ethics Statement

The studies involving human participants were reviewed and approved by Committees of Medical Ethics at the Nanfang Hospital, the Chinese PLA General Hospital, and the Lanzhou University Second Hospital. Written informed consent to participate in this study was provided by the participants’ legal guardian/next of kin. Written informed consent was obtained from the individual(s), and minor(s)’ legal guardian/next of kin, for the publication of any potentially identifiable images or data included in this article.

## Author Contributions

Y-MZ collected and analyzed data and drafted the main manuscript. Y-FL and Y-LL collected and processed the data. QL and X-PL supervised and conceptualized the study. XG and PD designed and conceptualized the study, interpreted the data, and revised and finalized the manuscript. All authors read and approved the final manuscript.

## Conflict of Interest

The authors declare that the research was conducted in the absence of any commercial or financial relationships that could be construed as a potential conflict of interest.

## Publisher’s Note

All claims expressed in this article are solely those of the authors and do not necessarily represent those of their affiliated organizations, or those of the publisher, the editors and the reviewers. Any product that may be evaluated in this article, or claim that may be made by its manufacturer, is not guaranteed or endorsed by the publisher.

## Footnotes

1
http://bio-bwa.sourceforge.net/

2
https://pymol.org/2/

## Supplementary Material

Supplementary Table 1 The 159 deafness-related nuclear genes.

Supplementary Table 2 Six mitochondrial deafness-related regions.

Supplementary Table 3 Three deafness-related miRNAs.

Supplementary Table 4 STR analysis of the affected twin brothers (II-3 and II-4).

Supplementary Table 5 Filtered variants of targeted NGS.

Supplementary Table 6 Likely pathogenic variants of targeted NGS.

Supplementary Table 7 Filtered variants of WES.

Supplementary Table 8 Likely pathogenic variants of WES.
