# Supplementary material for: Familial Temperature-Sensitive Auditory Neuropathy: Distinctive Clinical Courses Caused by Variants of the OTOF Gene
Source: Front Cell Dev Biol. 2021 Oct 7;9:732930. doi: 10.3389/fcell.2021.732930 (PMC8529165; doi:10.3389/fcell.2021.732930)
Supplement: Supplementary Table 1 — The 159 deafness-related nuclear genes. [file Table_1.docx]

**Supplementary Tables**

Table S1 The 159 deafness-related nuclear genes.

| *ACTG1* | *ADGRV1* | *ALX3* | *BSND* | *CABP2* | *CCDC50* | *CDH23* |
| --- | --- | --- | --- | --- | --- | --- |
| *CEACAM16* | *CHD7* | *CIB2* | *CLDN14* | *CLPP* | *CLRN1* | *COCH* |
| *COL11A1* | *COL11A2* | *COL1A1* | *COL1A2* | *COL2A1* | *COL4A3* | *COL4A4* |
| *COL4A5* | *COL4A6* | *COL9A1* | *COL9A2* | *CRYM* | *DFNA5* | *WHRN* |
| *DFNB59* | *DIABLO* | *DIAPH1* | *DIAPH3* | *DSPP* | *ECM1* | *EDN3* |
| *EDNRB* | *ELMOD3* | *ESPN* | *ESRRB* | *EYA1* | *EYA4* | *FGF3* |
| *FGF8* | *FGFR1* | *FGFR3* | *FLNA* | *FOXI1* | *FREM1* | *FXN* |
| *GATA3* | *GIPC3* | *GJB1* | *GJB2* | *GJB3* | *GJB6* | *GLYAT* |
| *GPSM2* | *GRHL2* | *GRXCR1* | *HARS* | *HARS2* | *HGF* | *HMX1* |
| *HOXA2* | *HSD17B4* | *IL13* | *ILDR1* | *KARS* | *KCNE1* | *KCNJ10* |
| *KCNQ1* | *KCNQ4* | *KITLG* | *KRT9* | *LAMA3* | *LARS2* | *LHFPL5* |
| *LOXHD1* | *LRTOMT* | *MARVELD2* | *MIR96* | *MITF* | *MPZ* | *MSRB3* |
| *MYH14* | *MYH9* | *MYO15A* | *MYO1A* | *MYO1E* | *MYO3A* | *MYO6* |
| *MYO7A* | *NDP* | *NDRG1* | *NEFL* | *NELL2* | *NF2* | *OPA1* |
| *OTOA* | *OTOF* | *OTOG* | *OTOGL* | *P2RX2* | *PABPN1* | *PAX3* |
| *PCDH15* | *PCDH9* | *PDZD7* | *PMP22* | *PNPT1* | *POLR1C* | *POLR1D* |
| *POU3F4* | *POU4F3* | *PROK2* | *PROKR2* | *PRPS1* | *PTPN11* | *PTPRQ* |
| *PTPRR* | *RDX* | *RPGR* | *SALL1* | *SALL4* | *SEC23A* | *SEMA3E* |
| *SERPINB6* | *SIX1* | *SIX5* | *SLC17A8* | *SLC19A2* | *SLC26A4* | *SLC26A5* |
| *SMAD4* | *SMPX* | *SNAI2* | *SOX10* | *STRC* | *TBC1D24* | *TCIRG1* |
| *TCOF1* | *TECTA* | *TIMM8A* | *TJP2* | *TMC1* | *TMEM126A* | *TMIE* |
| *TMPRSS3* | *TMPRSS4* | *TNC* | *TPRN* | *TRIOBP* | *TRMU* | *TSPEAR* |
| *TYR* | *USH1C* | *USH1G* | *USH2A* | *WFS1* |  |  |

Table S2 Six mitochondrial deafness-related regions.

| Locus | Location (RefSeq: NC_012920.1) |
| --- | --- |
| MT-RNR1 | chrM:648-1601 |
| MT-TL1 | chrM:3230-3304 |
| MT-CO1 | chrM:5904-7445 |
| MT-TS1 | chrM:7446-7514 |
| MT-TK | chrM:8295-8364 |
| MT-TE | chrM:14674-14742 |

Table S3 Three deafness-related miRNAs.

| Name | Chromosome Position (hg19) |
| --- | --- |
| miR-96 | chr7: 129414532-129414609 |
| miR-182 | chr7: 129410223-129410332 |
| miR-183 | chr7: 129414745-129414854 |

Table S4 STR analysis of the affected twin brothers (II-3 and II-4).

|  | II-3 | |  | II-4 | |
| --- | --- | --- | --- | --- | --- |
|  | Allele 1 | Allele 2 |  | Allele 1 | Allele 2 |
| Amelogenin | X | Y |  | X | Y |
| D3S1358 | 15 | 16 |  | 15 | 16 |
| D13S317 | 11 | 13 |  | 11 | 13 |
| D7S820 | 8 | 11 |  | 8 | 11 |
| D16S539 | 12 | 13 |  | 12 | 13 |
| Penta E | 14 | 19 |  | 14 | 19 |
| TPOX | 8 | 8 |  | 8 | 8 |
| TH01 | 6 | 9 |  | 6 | 9 |
| D2S1338 | 24 | 26 |  | 24 | 26 |
| CSF1PO | 7 | 12 |  | 7 | 12 |
| D19S433 | 13 | 15 |  | 13 | 15 |
| vWA | 16 | 17 |  | 16 | 17 |
| D5S818 | 11 | 11 |  | 11 | 11 |
| FGA | 22 | 23 |  | 22 | 23 |
| D6S1043 | 13 | 17 |  | 13 | 17 |
| D8S1179 | 13 | 16 |  | 13 | 16 |
| D21S11 | 25 | 29.2 |  | 25 | 29.2 |
| D18S51 | 13 | 13 |  | 13 | 13 |
